# Longitudinal micro-CT provides biomarkers of lung disease that can be used to assess the effect of therapy in preclinical mouse models, and reveal compensatory changes in lung volume

**DOI:** 10.1242/dmm.020321

**Published:** 2016-01-01

**Authors:** Greetje Vande Velde, Jennifer Poelmans, Ellen De Langhe, Amy Hillen, Jeroen Vanoirbeek, Uwe Himmelreich, Rik J. Lories

**Affiliations:** 1 Biomedical MRI/MoSAIC, Department ofImaging and Pathology, KU Leuven, B-3000 Leuven, Flanders, Belgium; 2 Laboratory of Tissue Homeostasis and Disease, Skeletal Biology and Engineering Research Center, Departmentof Development and Regeneration, KU Leuven, B-3000 Leuven, Flanders, Belgium; 3 Division of Rheumatology, University Hospitals Leuven, B-3000 Leuven, Flanders, Belgium; 4 Centre for Environment and Health, Department of Public Health and Primary Care, KU Leuven, B-3000 Leuven, Flanders, Belgium

**Keywords:** Quantification, Pulmonary fibrosis, Lung inflammation, Infectious diseases, Micro-computed tomography, *In vivo*, Lung volume, Disease models, Biomarkers, Bleomycin, Aspergillosis, Cryptococcosis

## Abstract

*In vivo* lung micro-computed tomography (micro-CT) is being increasingly embraced in pulmonary research because it provides longitudinal information on dynamic disease processes in a field in which *ex vivo* assessment of experimental disease models is still the gold standard. To optimize the quantitative monitoring of progression and therapy of lung diseases, we evaluated longitudinal changes in four different micro-CT-derived biomarkers [aerated lung volume, lung tissue (including lesions) volume, total lung volume and mean lung density], describing normal development, lung infections, inflammation, fibrosis and therapy. Free-breathing mice underwent micro-CT before and repeatedly after induction of lung disease (bleomycin-induced fibrosis, invasive pulmonary aspergillosis, pulmonary cryptococcosis) and therapy (imatinib). The four lung biomarkers were quantified. After the last time point, we performed pulmonary function tests and isolated the lungs for histology. None of the biomarkers remained stable during longitudinal follow-up of adult healthy mouse lungs, implying that biomarkers should be compared with age-matched controls upon intervention. Early inflammation and progressive fibrosis led to a substantial increase in total lung volume, which affects the interpretation of aerated lung volume, tissue volume and mean lung density measures. Upon treatment of fibrotic lung disease, the improvement in aerated lung volume and function was not accompanied by a normalization of the increased total lung volume. Significantly enlarged lungs were also present in models of rapidly and slowly progressing lung infections. The data suggest that total lung volume changes could partly reflect a compensatory mechanism that occurs during disease progression in mice. Our findings underscore the importance of quantifying total lung volume in addition to aerated lung or lesion volumes to accurately document growth and potential compensatory mechanisms in mouse models of lung disease, in order to fully describe and understand dynamic processes during lung disease onset, progression and therapy. This is highly relevant for the translation of therapy evaluation results from preclinical studies to human patients.

## Abstract (precis)

**Summary:** Quantifying not only aerated lung volume or lesion volumes but also the total lung volume from micro-CT is essential to document growth as well as potential compensatory mechanisms in the evaluation of mouse models of lung diseases and their therapy.

## INTRODUCTION

Obstructive, interstitial, infectious and malignant lung diseases remain among the most important health challenges, and novel or improved therapeutic strategies are essential for their treatment (WHO, 2014). Researchers heavily rely on the use of experimental animal models to gain new insights into pathogenesis, and to discover and validate specific therapeutic targets. Currently, histopathological analysis of lung tissues remains the gold standard for the assessment of preclinical models. Although histopathology and other *ex vivo* approaches offer plenty of opportunities to perform detailed molecular and cellular analyses, they are *a priori* limited to one measurement, providing only a snapshot of processes that are dynamic in time and space. *In vivo* micro-computed tomography (micro-CT) of the lung is therefore increasingly embraced in the field because it captures longitudinal information from individual animals, thereby reducing and refining the experimental setups (Gammon et al., 2014).

Providing excellent air-tissue contrast, micro-CT can be used to longitudinally evaluate disease progression and therapy effects in numerous models of lung diseases, including those of cancer, fibrosis, emphysema and transplantation (De Langhe et al., 2012; Li et al., 2013; Plathow et al., 2004; Wielputz et al., 2011; Wurnig et al., 2014). Lung micro-CT delivers visual and quantitative three-dimensional information about the whole lung – including regional differences – with high resolution and sensitivity, yielding translational data that align well with imaging assessments routinely performed in lung disease patients. Moreover, technological advances are translatable from and to the clinic. The different quantitative measures derived from a single lung scan can provide insight into lung anatomy, function and pathology.

Quantitative measurements, such as the mean lung density, volume of the lungs, air spaces or lesions, quantified from micro-CT images have been validated against gold standard histological measurements in specific lung disease models. For instance, quantification of progressive changes in the volume of air in the lung from micro-CT data was validated to correlate with progressive lung fibrosis and emphysema (De Langhe et al., 2012; Rodt et al., 2010). A segmentation-based and fully automated algorithm was proposed, resulting in efficient and user-independent quantification (De Langhe et al., 2012). The aerated lung volume can alternatively be obtained via an algorithm based on region growing, upon manual placement of a seeding point (Rodt et al., 2010). The amount of air within the lungs is the parameter that probably best corresponds to the total lung capacity (TLC) measured by pulmonary function tests. Nevertheless, diseases not only affect the air content of the lungs [represented by the hypointense (dark) voxels in micro-CT images], but can also increase lung ‘tissue’ (including lesions) volume, for instance in fibrotic disease, cancer, inflammation and infection. A semi-automated delineation approach was proposed for quantification of tumor mass from micro-CT images of a lung cancer mouse model (Li et al., 2013). However, lesions are not always defined by clear boundaries, complicating contouring of the hyperintense voxels and hence calculation of their volume. To circumvent this, the complete lungs can be [manually or (semi-)automatically] delineated to quantify the volume of hyperintense signals via overall thresholding within the total lung. Another approach is to calculate the mean density for all voxels in the volume of interest (VOI) defined by the lung. The mean lung density represents as such the bulk changes in air and tissue (including lesions) content of the lung as a whole. This approach was validated to provide an accurate readout for the extent of diffuse pathology such as lung fibrosis as detected by different magnetic resonance imaging (MRI) protocols (Vande Velde et al., 2014). Defining a VOI that comprises the whole lung also renders a value for the total lung volume, but there are no reports on the use of this biomarker to describe lung disease progression.

Despite these reports and the extent of validation performed when comparing quantitative imaging outcomes with histology assessments, many questions remain, in particular related to the choice of the optimal parameter(s) to address a specific research question. Moreover, how these different biomarkers compare and change throughout normal growth versus lung disease progression in rodent models remains largely unexplored.

Our working hypothesis is that micro-CT provides, in a non-invasive manner, several relevant biomarkers to describe how different aspects of the lung change during health, disease and therapy in animal models of pulmonary diseases.

In this work, we evaluate four different established and newly proposed quantitative lung parameters [aerated lung volume, lung tissue (including lesions) volume, total lung volume and mean lung density], quantified from repetitive *in vivo* micro-CT scans of freely breathing, anesthetized mice, to evaluate how these biomarkers are affected by the normal growth of the rodent and specifically how these changes affect the definition of control conditions during the course of a longitudinal experiment. We investigated which biomarkers reflect improvement of bleomycin-induced lung pathology and function upon treatment. To further evaluate the (timing of the) occurrence and importance of relevant changes in biomarkers associated with lung disease, we quantified lung volume changes for rapidly and slowly progressing mouse models of fungal lung infection.

## RESULTS

### Quantification of lung biomarkers for healthy adult mice reveals significant longitudinal changes

**Fig. 1.**
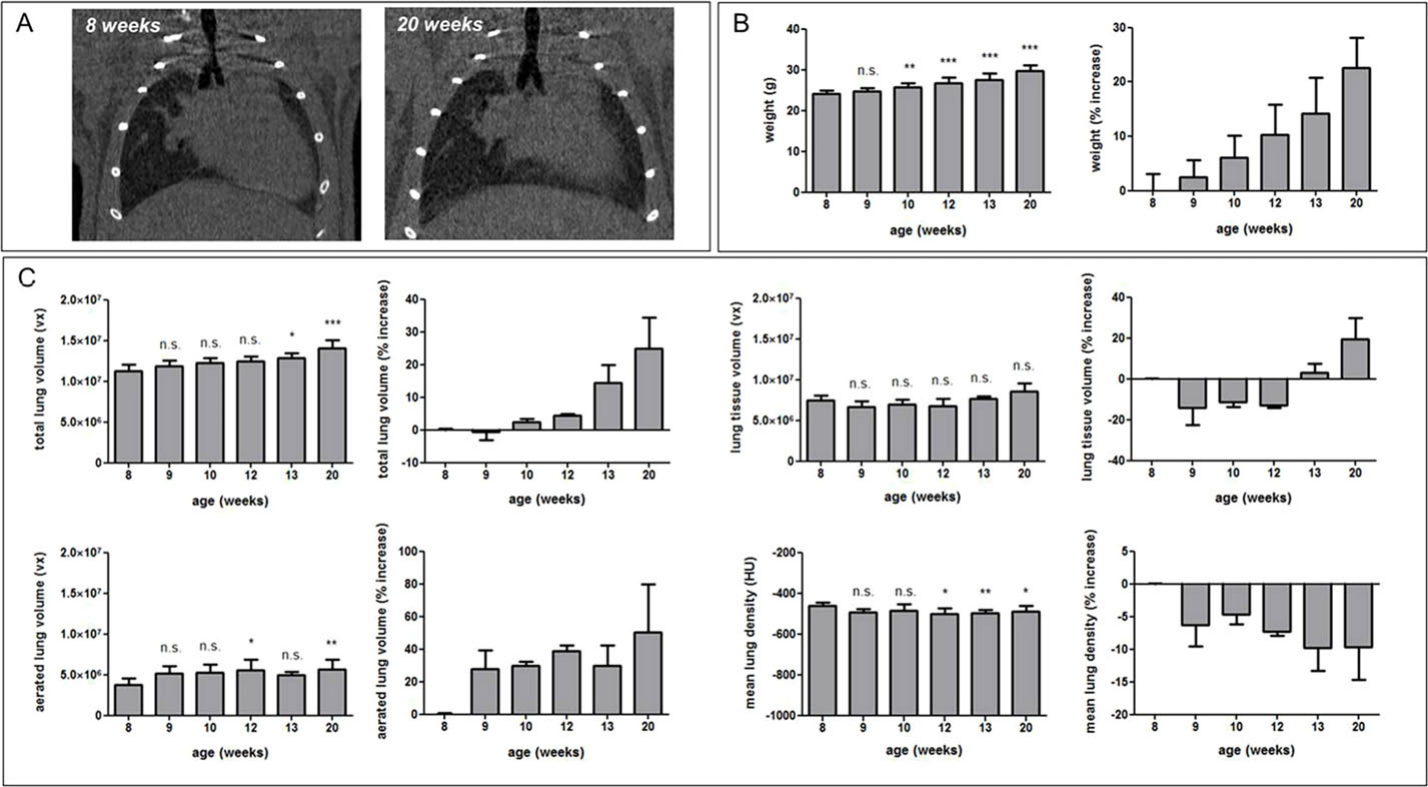
**Longitudinal *in vivo* lung micro-CT-derived biomarkers for healthy mice.** (A) Coronal micro-CT images of a healthy C57BL/6 mouse scanned at 8 and 20 weeks of age. (B) Graphs of the absolute weight and the increase in weight relative to 8-week-old mice for C57BL/6 mice of 8, 9, 10, 12, 13 and 20 weeks of age. (C) Graphs of the absolute value and increase in that value relative to 8-week-old mice, quantified from the longitudinal micro-CT images of healthy C57BL/6 mice, scanned at 8, 9, 10, 12, 13 and 20 weeks of age, for the following lung biomarkers: total lung volume, aerated lung volume, lung tissue volume and mean lung density. vx, voxels. Error bars indicate s.d. of replicate samples, *n*=3-10; *P<0.05, **P<0.01, ***P<0.005, n.s., not significant.

To evaluate how normal growth-associated changes would be reflected in the four different micro-CT-derived lung biomarkers, healthy adult C57BL/6 mice were weighed and scanned with micro-CT at 8, 9, 10, 12, 13 and 20 weeks of age (Fig. 1A). Eight-week-old adult mice showed steady weight gain with increasing age, which became significant as early as after 2 weeks (Fig. 1B). Twenty-week-old C57BL/6 mice weighed 22.60±5.62% more than they did at 8 weeks of age. The total lung volume followed the same evolution, resulting in a highly significant 25.00±9.43% increase in total lung volume at 20 weeks compared to 8 weeks of age (Fig. 1A,C). Further breakdown of this increase in lung volume into the contributions from the air and tissue compartments suggested that the increase in total lung volume was mainly due to an increase in aerated lung volume, whereas the lung tissue volume at first relatively decreased but started to increase around 13 weeks of age, along with an increase in total lung volume (Fig. 1C). These changes in lung air and tissue volumes had an effect on the mean lung density that showed an overall significant decrease of up to 7.83±4.61% at 20 weeks of age (Fig. 1C). Lung histology after the last imaging time point showed no evidence of any pathological (such as inflammatory), cellular or structural abnormalities in the lung tissue (Fig. S1), confirming the apparent healthy status of these mouse lungs. Similarly, microscopically detectable changes due to repeated micro-CT scanning of these mice could be excluded, supported by a previous study of the safety of longitudinal mouse lung micro-CT (Vande Velde et al., 2015), thereby minimizing the risk of any accidental pathology that could additionally underlie the observed growth-related changes in micro-CT-derived lung biomarkers for these healthy control mice.

### Quantification of lung biomarkers reveals a large increase in total lung volume for mice developing bleomycin-induced lung fibrosis

**Fig. 2.**
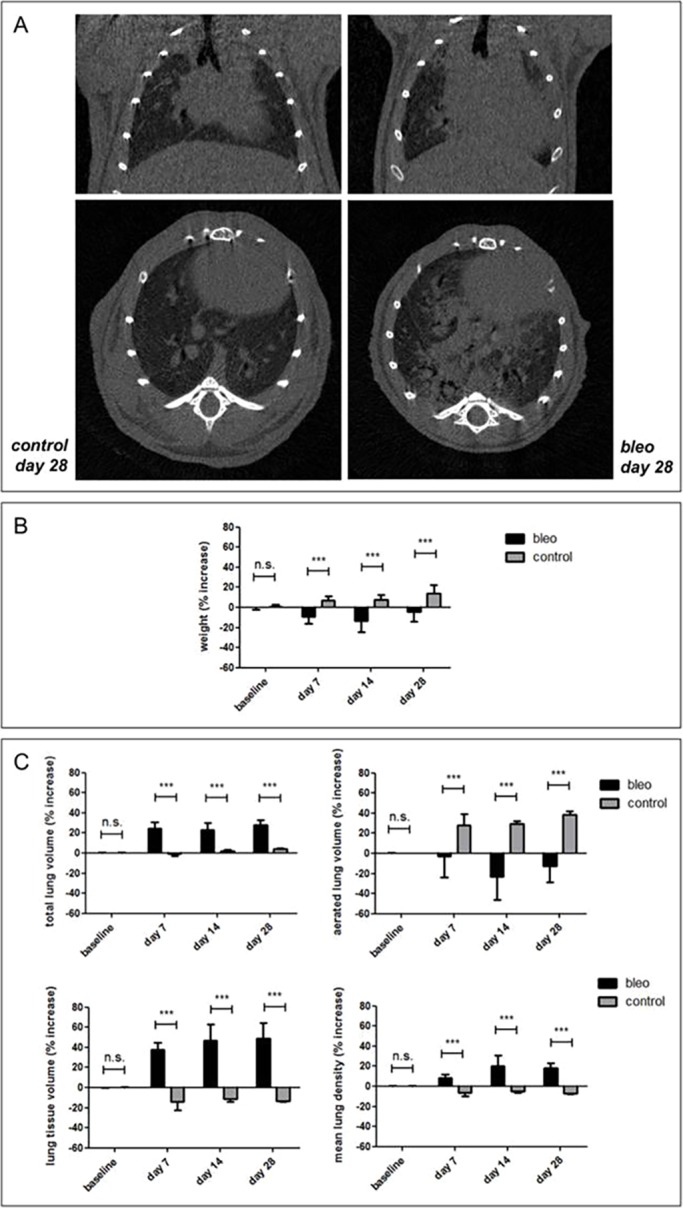
**Longitudinal *in vivo* lung micro-CT-derived biomarkers for mice with bleomycin-induced lung pathology.** (A) Coronal (top) and transverse (bottom) micro-CT images acquired from C57BL/6 mice at 28 days after PBS (‘control’, left panels) and bleomycin (‘bleo’, right panels) instillation. (B) Graph of the increase in weight relative to baseline for C57BL/6 mice before (baseline) and 7, 14 and 28 days after bleomycin (‘bleo’) or PBS (‘control’) instillation. (C) Graphs of the increase in value relative to baseline, quantified from the longitudinal micro-CT scans of bleomycin- (‘bleo’) or PBS- (‘control’) instilled mice for the following lung biomarkers: total lung volume, aerated lung volume, lung tissue volume and mean lung density. Error bars indicate s.d. of replicate samples, *n*=7 (5 at day 28) for bleomycin-instilled mice and *n*=3 for controls; ***P<0.005, n.s., not significant.

To evaluate how and to what extent lung inflammation and fibrosis induces changes in the lung, we quantified the four lung biomarkers from micro-CT scans that were acquired before and 7, 14 and 28 days after bleomycin instillation, and compared them with measurements for age-matched control mice (PBS-instilled; Fig. 2A). Bleomycin instillation in the lungs induces early inflammation that progresses into chronic lung fibrosis (Degryse et al., 2010; Hay et al., 1991) (Fig. S2). Animals suffering from bleomycin-induced lung disease showed significant weight loss compared to baseline and to their age-matched controls (Fig. 2B). Despite a weight loss in the order of 10-20% compared to baseline, bleomycin-instilled animals showed a marked and highly significant increase in total lung volume [24.14±6.69% at day 7 post instillation (p.i.) up to 28.25±5.32% at day 28 p.i.] already from the first week after bleomycin instillation compared to the age-associated increase in total lung volume of their age-matched controls (Fig. 2C). The progressive decrease in aerated lung volume and increase in lung tissue volume observed for bleomycin-instilled animals is consistent with progressive bleomycin-induced lung pathology. In contrast, control animals showed the opposite evolution for these biomarkers, i.e. a pronounced increase in aerated lung volume and a decrease in lung tissue volume over time (Fig. 2C). Mean lung density increased over time after instillation for bleomycin-induced animals, whereas it decreased for PBS-instilled controls (Fig. 2C).

### Imatinib treatment results in improved aerated lung volume and lung function but not in normalization of total lung volume

**Fig. 3.**
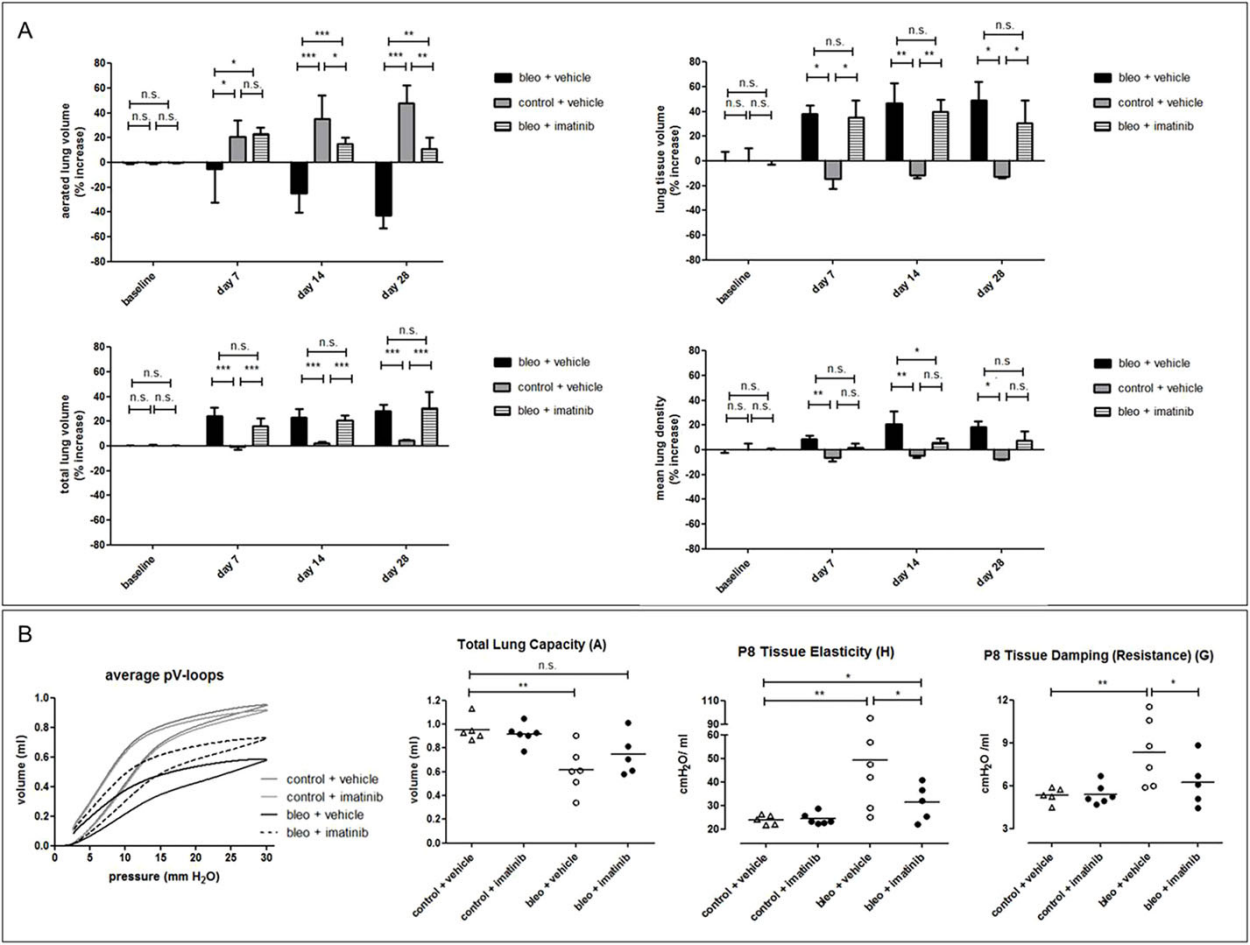
**Longitudinal *in vivo* lung micro-CT-derived biomarkers for bleomycin-induced mice upon treatment with imatinib.** (A) Graphs of the changes in lung biomarkers relative to baseline, quantified from the longitudinal micro-CT scans acquired at baseline and at 7, 14 and 28 days p.i. for bleomycin-instilled (‘bleo’) or PBS-instilled (‘control’, *n*=3) C57BL/6 mice, treated with imatinib (*n*=6) or vehicle (*n*=6). (B) Graphs of pulmonary function test results for the average PV loops (*n*=4-6 per curve), total lung capacity, tissue elasticity and damping (resistance). Error bars indicate s.d. of replicate samples; *P<0.05, **P<0.01, ***P<0.005, n.s., not significant.

To explore whether, and to what extent, therapeutic treatment would be reflected in the different lung biomarkers and particularly in total lung volume changes, we quantified the effect of imatinib, demonstrated previously to have a modest beneficial effect on bleomycin-induced lung disease as quantified by the aerated lung volume (De Langhe et al., 2012). Although, at 7, 14 and 28 days p.i., the imatinib-treated animals showed a significantly improved aerated lung volume compared to sham-treated bleomycin-induced mice, consistent with a decreased lung tissue volume, their total lung volume remained equally enlarged compared to baseline and controls (Fig. 3A). The mean lung density values for the imatinib-treated mice improved (decreased) over time, reflecting the bulk changes on air and tissue content of the lungs as a whole upon treatment (Fig. 3A). The improvement in terms of the increased aerated lung volume, which was not accompanied by a normalization of total lung volume (resulting in a net increase of air in the lungs), was reflected by improved volumetric measures of lung function, i.e. a clearly improved average pressure-volume (PV) loop and an increased functional total lung capacity of imatinib-treated mice (Fig. 3B). The significantly improved tissue elasticity and tissue resistance measures supported the overall lung function improvement upon imatinib treatment (Fig. 3B).

### Total lung volume changes are also present in disease models of rapidly and slowly progressing fungal lung infection

**Fig. 4.**
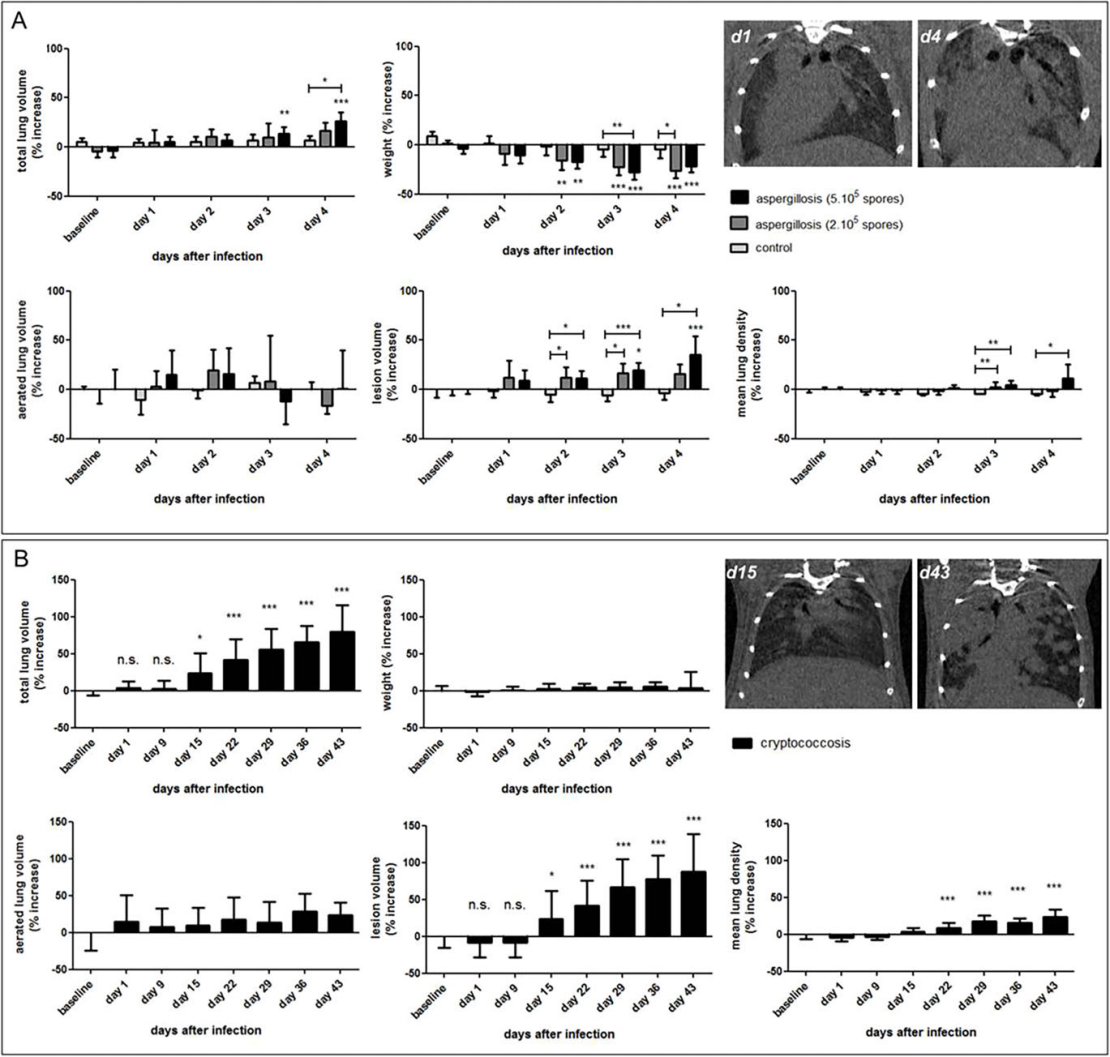
**Longitudinal *in vivo* lung biomarkers quantified for mice with rapidly and slowly progressing fungal lung infections.** (A) Graphs of the changes in lung biomarkers (quantified from the longitudinal micro-CT data) and weight relative to baseline, for immunosuppressed BALB/c mice at baseline and at 1, 2, 3 and 4 days p.i. with saline (‘control’, *n*=5), 200,000 [*n*=6 (3 at day 4)] or 500,000 [*n*=8 (3 at day 4)] *A. fumigatus* spores (‘aspergillosis’). Transverse micro-CT images for the same mouse are shown at 1 and 4 days after instillation of 500,000 *A. fumigatus* spores. (B) Graphs of the increase in lung biomarkers (quantified from the longitudinal micro-CT data) and weight relative to baseline, for immunocompetent BALB/c mice at baseline and at 1, 9, 15, 22, 29, 36 and 43 days p.i. with 50,000 CFUs of *C. gattii* (‘cryptococcosis’; *n*=24-30). Transverse micro-CT images are shown for the same mouse at 15 and 43 days after instillation of *C. gattii*. Error bars indicate s.d. of replicate samples; *P<0.05, **P<0.01, ***P<0.005, n.s., not significant.

Based on our previous results, we set out to further assess the occurrence, extent and relevance of total lung volume changes in other models of lung disease that are different from lung fibrosis in pathology and develop over different time scales. We therefore evaluated the longitudinal micro-CT scans acquired from models of pulmonary infection, namely mice that were induced to develop a rapidly progressing fungal lung infection (invasive pulmonary aspergillosis) within 4 days, or a slowly progressing fungal lung infection (pulmonary cryptococcosis) over a time span of 6 weeks. Quantification of the total lung volume, and its increase after 1, 2, 3 and 4 days of infection with *Aspergillus fumigatus*, showed a total lung volume increase from baseline to day 4 of 16% (inoculation with 2×10^5^ spores) up to 35% (inoculation with 5×10^5^ spores). Despite the animals' weight loss over time, lung parameters corresponded with the extent of disease as indicated by the initial inoculum size and tissue volume (including fungal mass) (Fig. 4A; Fig. S3A,B). The weight loss of the control mice reflected their general immunosuppressed state. Mice infected with a small inoculum of *Cryptococcus gattii* gradually developed a very slowly progressing lung infection of which the first signs did not appear on the micro-CT scans until 2 weeks after infection. The total lung volume for these animals steadily increased, reaching significantly different values from baseline at 2 weeks after infection onwards (Fig. 4B). The average increase after 6 weeks was 80%, with peaks up to 130% compared to baseline values (data not shown), also mirroring the increase in fungal mass. However, the aerated lung volume and physical appearance of the animals remained unchanged (Fig. 4B; Fig. S3C,D). During these 6 weeks of progressive lung infection, these mice showed no phenotypical signs of disease, as also supported by the gradual increase in their weight with age (Fig. 4B). The mean lung density increased with progressive lung cryptococcosis, corresponding to the overall increased fungal burden in the lung.

## DISCUSSION

Non-invasive lung micro-CT provides a valuable tool to acquire longitudinal information on lung disease onset, progression and subsequent therapy evaluation in preclinical models, which is essential for improving our understanding of dynamic processes involved in pathogenesis and treatment of lung diseases. In this study, we investigated how this longitudinal information can be quantified through several micro-CT-derived biomarkers that describe different aspects of lung disease. To answer the question  of which parameter(s) should best be used to describe meaningful changes in the lung, we investigated how total lung volume, aerated lung volume, lung tissue volume and mean lung density change over time for healthy mice, different types of lung disease models and upon their treatment. To this end, we employed a high-resolution respiratory gated 4D micro-CT protocol with a nevertheless short scanning time that can be repeatedly used in free-breathing mice (De Langhe et al., 2012). Such a non-invasive approach is highly relevant when working with lung disease models because these animals would suffer from the repeated intubations applied for breath-hold-gated or prospectively gated micro-CT protocols (Artaechevarria et al., 2010; Badea et al., 2004); this approach allowed us to scan the animals on a weekly and even daily basis with zero mortality.

Here, we documented that the lungs of adult mice between the age of 8 and 20 weeks continuously grow, following a steady increase in body weight. We showed that this increase in total lung volume with age is mainly accompanied by an increase of aerated lung volume. The changes in aerated, tissue and total lung volume affect the values for the mean lung density, with none of the studied lung biomarkers remaining stable during longitudinal follow up of healthy mice. Because coincidental pathology (such as a respiratory infection) and potential effects from repeated micro-CT scanning could be excluded (Vande Velde et al., 2015), the observed changes in micro-CT-derived lung parameters can be considered to be exclusively age-related. As a consequence, absolute values should always be compared with age-matched controls when evaluating how lung biomarkers change after an intervention (disease induction, therapeutic intervention) rather than comparing each animal with its own baseline control, because values obtained after intervention would include an effect of both the intervention and of age, complicating interpretation of the longitudinal quantitative readouts. We assessed mice of 8 to 20 weeks of age because they are by far the most commonly used in preclinical research. Alternatively, one could consider using older mice because their lung biomarkers are less likely to change significantly from week to week. Yet, data on normal mouse lung development beyond the time frame of this study is scarce (Martorana et al., 1989; Pinkerton et al., 2014) and *in vivo* studies are lacking. Notwithstanding that, in more mature animals, the age-associated changes might become less pronounced, in particular when compared to more substantial disease-related changes. Therefore, longitudinal studies with appropriate statistical methods that include a repeated measurements assumption could still make reliable use of the animal's baseline data as a control, which would imply an additional reduction of the number of animals needed for a longitudinal study as compared to cross-sectional studies.

Next, we demonstrated that early inflammation and progressive fibrosis can be monitored not only by the decrease in air spaces (De Langhe et al., 2012) but also by micro-CT-derived biomarkers that reflect the increase in lesion lung tissue volume and the overall increase in mean lung density due to the induced pathology. Of note, our quantitative findings reveal a large and significant increase in total lung volume in the bleomycin-induced mouse model of lung fibrosis. This increase in total lung volume must be taken into account for the interpretation of the aerated lung volume, tissue volume and mean lung density measures in this model. Although the marked decline of aerated lung volume agrees with gold standard measurements of the extent of bleomycin-induced fibrosis and lung function (De Langhe et al., 2012), quantifying disease solely by the effect it has on the size of the air space underestimates disease progression (quantified by the ‘lung tissue volume’ biomarker) because of the substantial increase in total lung volume. The increase in total lung volume is larger than the decrease in aerated lung volume, but might – depending on the model studied – be partially caused by alveolar or airway distention due to accumulation of fluid. The total lung volume increase does not entirely compensate for the loss of airspaces caused by tissue deposition, which result in a net decrease of aerated lung volume and the subsequent worsening of the condition of the mice. A similar increase in total lung volume was quantified from parallel proton-density-, T_2_- and T_2_*-weighted respiratory-gated MRI scans that we acquired from these animals at expiration (G.V.V. et al., unpublished results), and was also recently reported for mice and rats scanned with MRI, albeit for a different bleomycin-induction model (Egger et al., 2014). Whereas aerated lung volume agrees very well with pulmonary function measurements (De Langhe et al., 2012), the mean lung density biomarker correlates even better with gold standard readouts for fibrosis and collagen deposition (Vande Velde et al., 2014).

Upon treatment of bleomycin-induced lung disease with imatinib, the improvement seen in aerated lung volume and lung function was not accompanied by a normalization of the progressively increased total lung volume. This finding suggests that some of the total lung volume changes reflect a compensatory mechanism and should probably not be considered a direct marker of disease (Egger et al., 2014). Total lung volume changes are more likely to be a consequence of the remodeling processes that precede them, and should as such better be regarded as an indirect parameter, whereas other measurements quantifying air content or lung tissue content are more likely to reflect more directly pathologic and therapeutic changes in the lungs.

Similar but smaller increases in lung volume occurred on a shorter time scale (i.e. within a few days) in mice with rapidly progressing invasive pulmonary aspergillosis. When an infectious disease develops more slowly, as seen in a mouse model of pulmonary cryptococcosis, at least part of the large total lung volume increase seems to compensate for the fungal growth to possibly maintain the aerated lung volume. This is consistent with the absence of clinical symptoms until week 5-7 after inoculation of the fungus, when the animals also become clinically ill.

Our findings underscore the importance of quantifying not only aerated lung volume or lesion volume but also the total lung volume to document growth as well as potential compensatory mechanisms in mouse models of lung disease. Up until now, such volume changes have been mainly overlooked, notwithstanding their importance for the quantification and interpretation of biomarkers that report on air and tissue content and mean lung density of the lung in the context of preclinical lung disease and therapy examinations as expounded here. We therefore recommend considering more than one if not all herein described lung biomarkers, in order to fully describe and better understand dynamic processes during lung disease onset, progression and therapy.

Some interesting questions remain. In general, it is tentative to speculate that part of the volume increase observed serves as a compensatory mechanism and, at least in the slowly progressing infection model, this suggests that the animal becomes ill when the compensatory increase in total lung volume fails, as seen by the net decrease in aerated lung volume for diseased animals. Nonetheless, volume increases, whether representing compensatory changes in lung volume or extension by pathological processes (such as collagen deposition, fungal growth etc.), need at least a couple of days to develop. That such changes need a few days to occur is also supported by the results of Egger et al. (2014). They showed that ovalbumin-induced inflammation after 24 and 48 h did not result in enlargement of the lungs (Egger et al., 2014), whereas we showed that early inflammation, the dominant process 1 week after bleomycin instillation, resulted in a 24% increase in total lung volume after 7 days.

It is important to keep in mind that an effect of repeated radiation exposure on the disease process itself cannot be excluded, but seems rather unlikely or very limited compared to the disease-related changes that we observed. Whereas the relatively low cumulative radiation dose of weekly repeated micro-CT scans fall well within a healthy mouse's repair capacity (Vande Velde et al., 2015), this is less clear for diseased lungs and in situations where daily micro-CT scans are warranted, such as for the aspergillosis model. In those cases, recent advances in lung MRI could provide an alternative that is devoid of any concerns regarding radiotoxic influences on the disease process (J.P., A.H., U.H., G.V.V. et al*.*, unpublished results; Vande Velde et al., 2014).

It remains unclear how these total lung volume changes in mouse models of disease relate to human lung disease. Although our current knowledge does not allow anything more than speculation concerning the underlying potential mechanism, something to consider is that, unlike in humans, the chest wall and other thoracic structures in mice are very compliant, giving them the ability to crawl through small holes (Irvin and Bates, 2003). It is not unlikely that this property of mouse lungs results in a different response to disease and therapy, such as the marked total lung volume increases we observed here. These questions warrant further research. We therefore underscore the importance of documenting total lung volume changes together with other longitudinal micro-CT-derived biomarkers in experimental lung studies from free-breathing animals as an indispensable step towards unraveling the underlying physiological mechanisms. Because we are dependent on animal models for preclinical therapy testing, this is relevant for patient care because the imperfections of mouse models mimicking human disease are likely to account for at least some of the many discrepancies found when translating therapy evaluation results from mouse studies to human patients (Moeller et al., 2008; Moore and Hogaboam, 2008). Characterizing thoroughly what happens in mouse models of lung disease can only be beneficial concerning the translatability of results from mice to men. Non-invasive, longitudinal imaging and accurate quantification of disease progression and the effects of therapy will likely be indispensable tools in this regard.

## MATERIALS AND METHODS

### Study design

To monitor longitudinal changes in the adult mouse lung during normal growth and different types of lung pathology (i.e. bleomycin-induced lung inflammation and fibrosis, pulmonary cryptococcosis, and invasive pulmonary aspergillosis) and treatment, all mice were scanned *in vivo* with micro-CT at baseline and at multiple time points after induction of the respective lung disease, with or without fibrosis treatment. After the last imaging time point, pulmonary function was tested to evaluate functional improvement upon therapy, mice were sacrificed and the lungs isolated for histology. To quantitatively describe longitudinal changes in the lungs due to growth, disease and therapy, we evaluated for all mice and mouse models the following four different biomarkers derived from the longitudinal micro-CT data: aerated lung volume, lung tissue volume, total lung volume and mean lung density.

### Animal models of lung disease

All animal experiments were carried out in compliance with national and European regulations and were approved by the animal ethics committee of KU Leuven. Male 8-week-old C57BL/6 mice (Janvier, Le Genest, France) were scanned without intervention and instilled intra-tracheally with bleomycin [0.05 U in 50 µl of phosphate-buffered saline (PBS); Sanofi-Aventis, Diegem, Belgium] to induce lung fibrosis, or underwent a sham procedure (50 µl of PBS) as described before (De Langhe et al., 2012; Vande Velde et al., 2014). A subgroup of mice was treated with imatinib dissolved in distilled water [50 mg/kg body weight/day intraperitoneally (i.p.); Novartis, Vilvoorde, Belgium] or with the vehicle alone as control. For induction of aspergillosis, 10-week-old BALB/c mice were immune-suppressed (cyclophosphamide i.p., 150 mg/kg body weight) on day 4 and 1 day prior to intranasal infection with 2×10^5^ or 5×10^5^ *A. fumigatus* (ASFU 1731) spores or sterile saline. For induction of cryptococcosis, immune-competent 8-week-old BALB/c mice were infected through inhalation of 50×10^3^ colony-forming units (CFUs) *C. gattii* (strain R265 described in Voelz et al., 2010) or PBS as control.

### Lung imaging

Mice were anesthetized with 1.5-2% isoflurane in 100% oxygen. Respiratory-gated micro-CT images of free-breathing animals were acquired using a small-animal micro-CT scanner (SkyScan 1076, Bruker microCT, Kontich, Belgium) according to a previously validated scan protocol with excellent reported reproducibility (De Langhe et al., 2012). Scan parameters were 50 kVp X-ray source voltage combined with a 0.5 mm aluminum filter, 180 µA current and 120 ms exposure time per projection, acquiring 9 projections per position with 0.7° increments over a total angle of 180°, resulting in a dose of 813 mGy (per scan) and scanning time of 12 min yielding 4D data, i.e. four reconstructed 3D datasets with 35 µm^3^ isotropic voxel size, corresponding to four phases of the breathing cycle. Repeated lung micro-CT using this protocol does not result in radiotoxic damage to the lungs (Vande Velde et al., 2015).

### Image analysis and quantification

Micro-CT data were retrospectively gated, reconstructed, visualized and processed using software provided by the manufacturer. For Hounsfield unit (HU) calibration, a phantom was scanned consisting of an air-filled 1.5 ml tube inside a water-filled 50 ml tube. Based on full stack histograms of a VOI containing only water or air, the mean gray scale index of water (81.32) was set to 0 HU and the gray scale index of air (0) to −1000 HU. Quantification of the aerated lung volume was performed using a custom-written fully automated algorithm that was applied to all datasets, as previously validated in De Langhe et al. (2012). Quantification of the total lung volume and mean lung density was done for a VOI covering the lung comprised of regions of interest (ROIs) that were manually delineated on the coronal images, thereby avoiding the heart and main blood vessels. Tissue signals should therefore be considered as for perfused tissue. Lung tissue volume is calculated as the total lung volume (manually delineated) minus the aerated lung volume (automatically derived). The retrospectively gated micro-CT data yields information on four different phases of the breathing cycle (i.e. end of inspiration, end of expiration and intermediate phases) of which tidal volume data can be derived. All data presented here correspond to the end-expiratory breathing phase.

### Pulmonary function tests

After the last micro-CT imaging session, pulmonary function tests were performed similarly to as described before (De Langhe et al., 2012). In brief, deeper anesthesia was achieved with an i.p. injection of pentobarbital (70 mg/kg body weight) (CEVA, Diegem, Belgium) to suppress spontaneous breathing. After a tracheostomy, the mice were connected to the flexiVent system (SCIREQ, Montreal, Canada). The computer-controlled small-animal ventilator ventilated the mice quasi-sinusoidally with a tidal volume of 10 ml/kg body weight at a frequency of 150 breaths/min and a positive end-expiratory pressure of 2 cm H_2_O to achieve a mean lung volume close to that during spontaneous breathing. Two perturbations were performed: a snapshot perturbation and a PV loop with constant increasing pressure (PVr-P). Each time before performing these perturbations, a total lung capacity perturbation (TLC) was carried out to normalize the lungs. The snapshot and PVr-P perturbations were performed until three acceptable measurements [coefficient of determination (COD)=0.95] were recorded in each individual mouse, of which an average was calculated. The snapshot perturbation was imposed to measure resistance, compliance, and elastance of the whole respiratory system (airways, lung and chest wall). The PVr-P perturbation generated vital (total) lung capacity (A), inspiratory capacity from zero pressure (B), form of deflating PV loop (K), static compliance (Cst), static elastance (Est), tissue damping (resistance, G) and hysteresis, until three acceptable measurements (COD=0.95) were recorded for each subject, of which an average was calculated. Raw data from the PVr-P perturbation was used to reconstruct the presented PV loops (group averages; *n*=4-6 per group).

### Histology

The left lung was collected for histopathology, inflated with 500 µl of 10% formalin in PBS via the left main bronchus and fixed in formalin for 24 h. After paraffin embedding, 5-µm sections were cut throughout the lung and stained with hematoxylin-eosin (HE) and/or periodic acid-Schiff (PAS) stains. Histopathological sections were examined using an Axioplan light microscope (Carl Zeiss, Oberkochen, Germany).

### Statistics

Data were analyzed using Prism 5.04 (Graphpad Software, San Diego, CA). *t*-tests or one- or two-way ANOVA with the appropriate post-hoc tests for multiple comparisons were used to evaluate the differences between two or more groups for every time point. Whenever a data point for an animal was missing (e.g. due to premature death or a technical issue), the longitudinal data for this animal was not included in the analysis. *P*-values <0.05 were considered significant. Data are presented as mean±s.d.
